# Supplementary material for: Extending the Host Range of Fusarium Poae Virus 1 from Fusarium poae to other Fusarium Species in the Field
Source: Viruses. 2022 Oct 13;14(10):2246. doi: 10.3390/v14102246 (PMC9610284; doi:10.3390/v14102246)
Supplement: Supplementary file 1 [file viruses-14-02246-s001.zip › Table S1.pdf]

Table S1. Primers and sequences used in this study.

Primer name	Sequence 5' → 3'
For virus amplification	
CP5UTRF1	TTGCTGCAATCGCTAAACGC
CP5UTRR1234	TCAGTGGGATCGCCATGAGA
CP5UTRF2	ATTGAATATTACTCTTCACTCTTAC
CP5UTRF3	GATGCCGTCGAACTCCGTAC
CP5UTRF4	TTGATGCCGTCGAACTCCGTAC
CP3UTRF1	CAAAGCAGGCACGAACGGACAT
CP3UTRR1	GGCGGAATGAAGCAATGAAG
CP3UTRF23	GCACGAACGGACATCTACTC
CP3UTRR2	AGCGAAAAACTATAATCGAGTTTCG
CP3UTRR3	AACATAGGGGAAAAACAATCAAAAG
CP-F	TCGATTCCTCACTCAACGTCCTA
CP-R	TGCAGATTGGAGGTACTGAGAAT
RDRP5UTRF1	TAAACAATCTCAACAAGGCTTC
RDRP5UTRR123	GAAGCCTTGTTGAGATTGTTTA
RDRP5UTRF2	GGGGACAAGTTTGTACAAAAAAGCAGGCTGAATTTCTCCAGCTTCGTGACC
RDRP5UTRF3	GGGGACCACTTTGTACAAGAAAGCTGGGTTGAAGCCGCCTGGCATCACTCG
RDRP-F	CACGGTTATGCTCCAACGTATGAG
RDRP-R	TAGCCAGGTAGGTGAGACTGAATG
For detection of *Fusarium* species	
Fg-16F	CTCCGGATATGTTGCGTCAA
Fg-16R	GGTAGGTATCCGACATGGCAA
ITS1	TCCGTAGGTGAACCTGCGG
ITS4	TCCTCCGCTTATTGATATGC
EF-1α-F	ATGGGTAAGGARGACAAGAC
EF-1α-R	GGARGTACCAGTSATCATGTT
For RT-PCR	
RdRp-RTF	CACGGTTATGCTCCAACGTATGAG
RdRp-RTR	TAGCCAGGTAGGTGAGACTGAATG
α-tubulin-F	GAGGATCTCGCTGCCCTTGA
α-tubulin-R	AGCAGGCTAAAACTGGTTCAAAG
