# Supplementary figures and images for: Extending the Host Range of Fusarium Poae Virus 1 from Fusarium poae to other Fusarium Species in the Field
Source: Viruses. 2022 Oct 13;14(10):2246. doi: 10.3390/v14102246 (PMC9610284; doi:10.3390/v14102246)

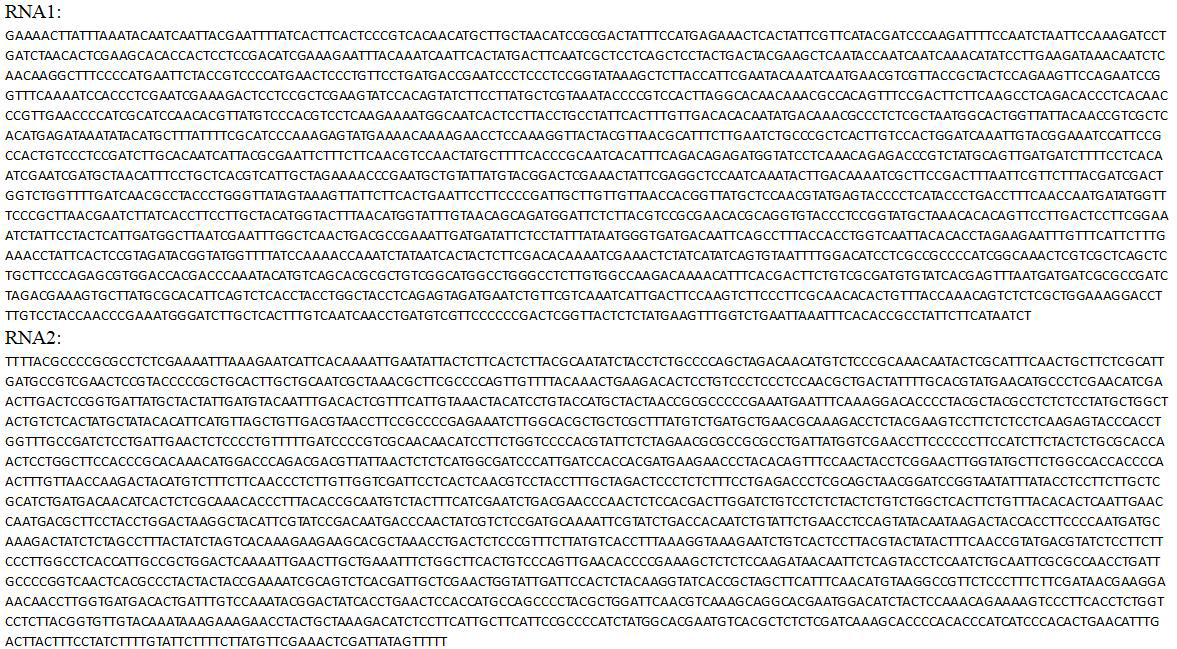

Supplement: Supplementary file 1 [file viruses-14-02246-s001.zip › FigureS1.tif]

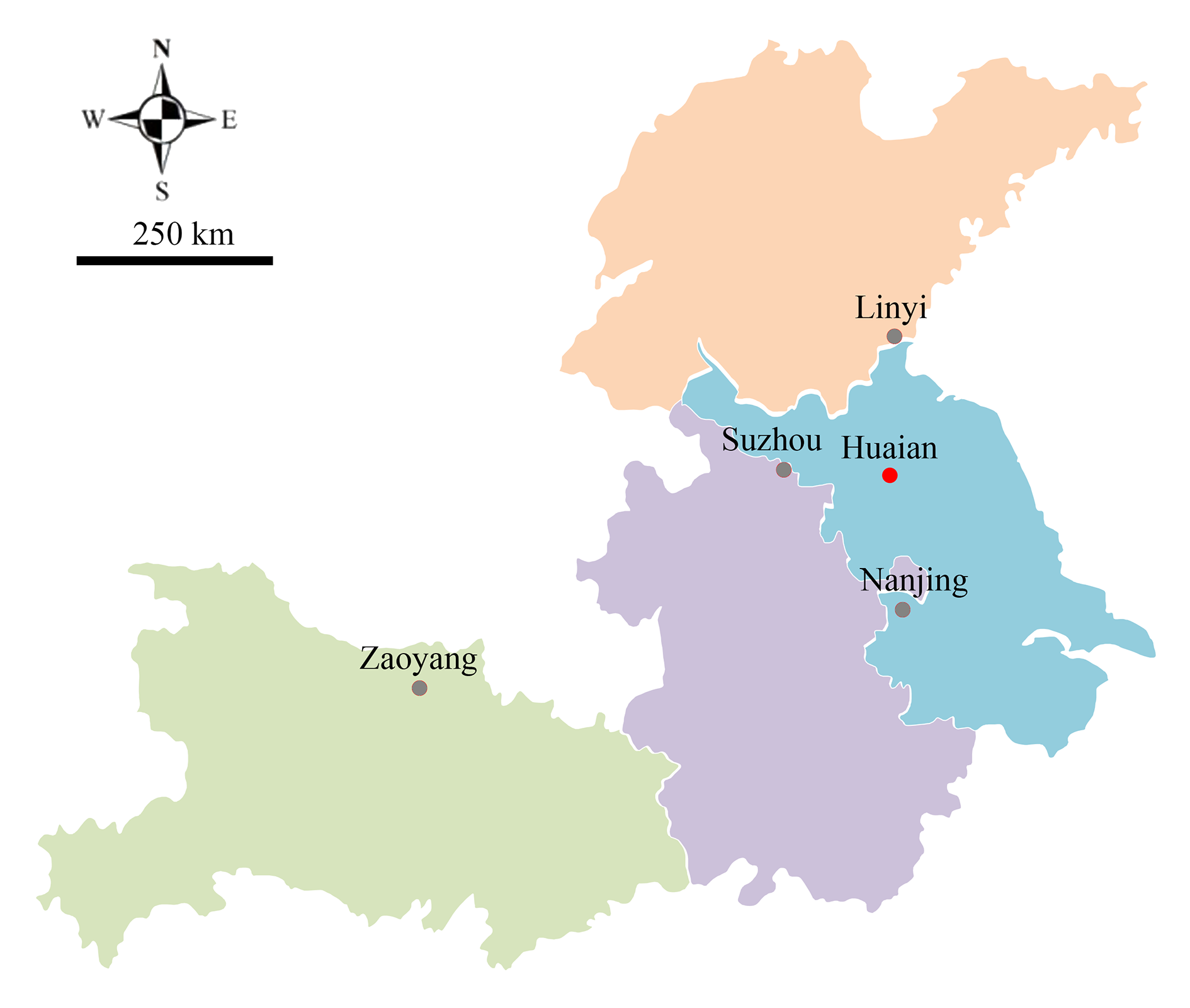

Supplement: Supplementary file 1 [file viruses-14-02246-s001.zip › FigureS2.tif]
